# Commentary: The correct answer is not true or false, but the ratio might be

**DOI:** 10.1016/j.xjon.2022.04.005

**Published:** 2022-04-13

**Authors:** Marvin D. Atkins, Michael J. Reardon

**Affiliations:** Department of Cardiovascular Surgery, Houston Methodist Hospital, Houston, Tex

**Figure:**
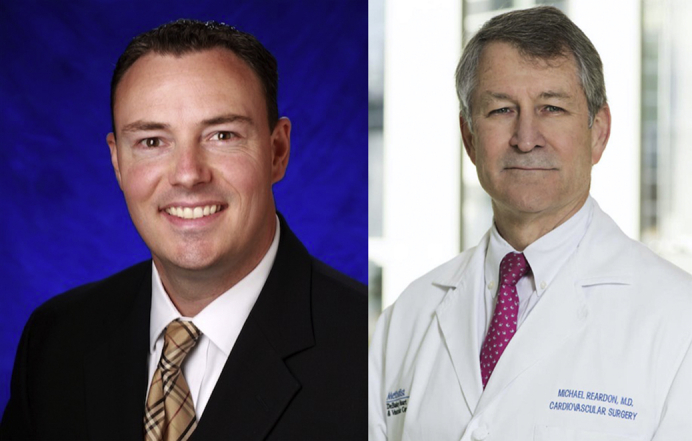
Marvin D. Atkins, MD, and Michael J. Reardon, MD

**Central Message**

Life-threatening events distal to the repair of acute type A aortic dissection remain a significant problem. Predicting those at risk for this may allow early intervention and improved outcomes.

See Article page 75.

Surgery for acute type A aortic dissection (ATAAD) has the goal of resection of the intimal entry tear, restoration of true lumen perfusion, and treatment of proximal cardioaortic complications. Although the immediate risks are prevented, the risk of late aortic events remains. The rate of distal aortic intervention varies. Roselli and colleagues^1^ from the Cleveland Clinic reported a probability of distal reintervention following ATAAD of 38% at 10 years. In contrast, the Nordic Consortium for Acute Type A Aortic Dissection (NORCAAD)^2^ reported their experience with reoperation following ATAAD and found freedom from the risk of distal reoperation was high, 96.9% at 5 years. The end point of aortic reoperation, however, is confounded by selection bias, as high-risk patients who have an indication for repair may not be offered surgery. Gaudino and colleagues^3^ performed a meta-analysis of 7821 patients from 47 studies with ATAAD repair. They found the rate of distal reoperation was 11.5% at a mean of 5.2 years. The truth likely resides somewhere in the middle. Prediction of the risk of distal aortic events is imperative. Previous studies have suggested a patent false lumen, intimal tear >1 cm in the proximal descending thoracic aorta, location of the intimal tear along the greater curvature, initial total aortic diameter of 40 mm, and an initial false lumen diameter of >22 mm are predictive of subsequent aneurysmal expansion.^4^

In this issue of the *Journal*, Igarashi and colleagues^5^ at Fukushima Medical University reviewed 74 patients at their hospital between 2001 and 2015 who underwent repair for ATAAD and had postoperative computed tomography scan data for comparison. Patients with known connective tissue disorders were excluded from the analysis. The aim of the study was to identify anatomical factors of the postoperative computed tomography scan associated with late aortic events such as reoperation or rupture. The authors surmised that the diameters and the ratio of the true and false lumen are a surrogate for increased pressure within the false lumen that left untreated ultimately leads to aneurysmal degeneration and rupture. They found that a ratio of false lumen diameter (FL-D) > true lumen diameter (TL-D) was significantly associated with late aortic events. The rate of freedom from late aortic events in the downstream aorta at 5 and 9 years was 81% and 60.7% in the FL-D > TL-D group and 92% and 88.6% in the FL-D < TL-D groups, respectively. The authors suggest that in patients with initial postoperative imaging showing a false lumen > true lumen ratio, earlier intervention might be warranted. Such interventions include thoracic endovascular aortic repair, coil embolization of the false lumen, or plugs to seal proximal or distal entry/re-entry tears.

The authors are to be congratulated for their contribution to our further understanding of such high-risk aortic features following proximal aortic repair that predispose to late aortic events. Whether earlier endovascular intervention in such patients with high-risk ATAAD is associated with improved aortic remodeling and/or a survival advantage remains to be known. The evidence defining those patients at high-risk for late aortic events continues to grow, and the aforementioned study contributes to our understanding.

## References

[1] Roselli E.E., Loor G., He J., Rafael A.E., Rajeswaran J., Houghtaling P.L. (2015). Distal aortic interventions after repair of ascending dissection: the argument for a more aggressive approach. J Thorac Cardiovasc Surg.

[2] Pan E., Gudbjartsson T., Ahisson A., Fuglsang S., Geirsson A., Hansson E.C. (2018). Low rates of reoperations after acute type A aortic dissection repair from the Nordic Consortium Registry. J Thorac Cardiovasc Surg.

[3] Gaudino M., Girardi L.N., Rahouma M., Leonard J.R., Di Franco A., Lau C. (2018). Aortic re-operation after replacement of the proximal aorta: a systematic review and meta-analysis. Eur J Vasc Endovasc Surg.

[4] Nienaber C.A., Kische S., Rousseau H., Eggebrecht H., Rehders T.C., Kundt G. (2013). Endovascular repair of Type B aortic dissection: long-term results of the randomized investigation of stent grafts in aortic dissection trial. Circ Cardiovasc Interv.

[5] Igarashi T., Sato Y., Satokawa H., Takase S., Iwai-Takano M., Seto Y. (2022). Ratio of the false lumen to the true lumen is associated with long-term prognosis after surgical repair of acute type A aortic dissection. J Thorac Cardiovasc Surg Open.

